# A Theoretical Investigation of Nonadiabatic Dynamics and Time-Resolved NEXAFS Spectra of Uracil

**DOI:** 10.1021/acsomega.6c06959

**Published:** 2026-09-08

**Authors:** Bonasree Roy, Evgenii Titov, Peter Saalfrank

**Affiliations:** 426790 University of Potsdam, Institute of Chemistry, Theoretical Chemistry, Karl-Liebknecht-Straße 24−25, Potsdam 14476, Germany

## Abstract

Uracil, a fundamental RNA nucleobase, exhibits complex excited-state dynamics governed by nonadiabatic processes that prevent photodamage. Upon photoexcitation, population initially reaches the bright ππ* (S_2_) state and subsequently relaxes to the dark nπ* (S_1_) state via conical intersections, before returning to the ground state on picosecond–nanosecond time scales. In this work, we investigate the ultrafast S_2_ → S_1_ relaxation pathway using time-dependent density functional theory (TD-DFT)-based nonadiabatic surface hopping dynamics in combination with simulated time-resolved near-edge X-ray absorption fine structure (TR-NEXAFS) spectroscopy of oxygen, nitrogen and carbon K-edges. This combined approach allows us to capture both electronic and nuclear motion during the ultrafast relaxation and to identify atom-specific contributions to the decay process. In particular, ππ* to nπ* transitions occurring within the first 140 fs leave traces in O 1s and C 1s NEXAFS signals, indicating C=O bond elongations and torsional motion along the C=C bond, respectively. Overall, our results provide a detailed picture of population transfer, electronic-state trapping, and structural evolution in photoexcited uracil.

## Graphical abstract

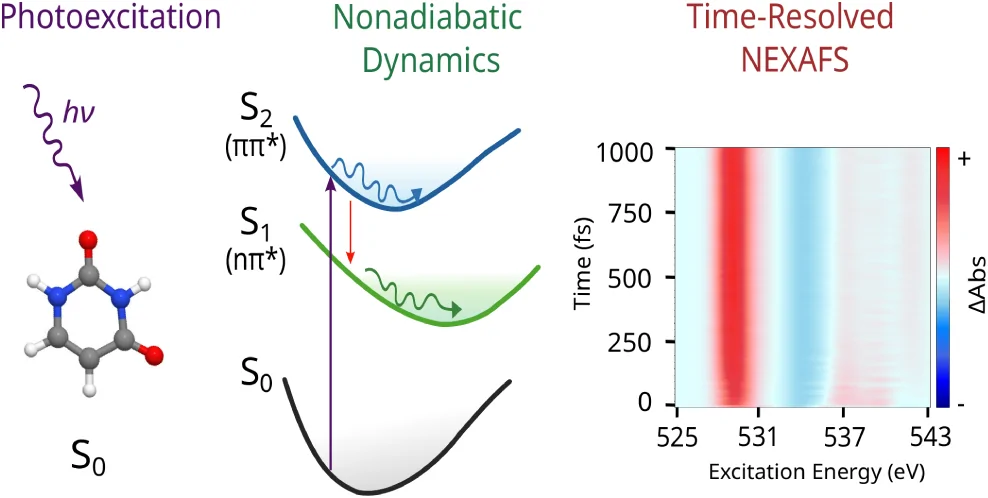

## 1 Introduction

The nucleobases have been the center of attraction for the study of molecular photodamage for several decades.^1–3^ Although nucleobases are vulnerable to photodamage and subsequent mutations in the ultraviolet and even vacuum–ultraviolet region,^4–6^ they rarely accumulate such damage. This is achieved by ultrafast relaxation to the ground state which provides the photostability to the molecules.^7–9^ Among these nucleobases, uracil has served as a model system for understanding RNA photochemistry due to its relatively simple structure and well-characterized photophysics.

Previous literature has shown that uracil in the gas phase decays through two main channels. One is the dominant indirect pathway, where the population undergoes rapid S_2_(ππ*) → S_1_(nπ*) internal conversion and becomes transiently trapped in the S_1_ state before returning to the triplet states and ultimately ground state on picosecond–nanosecond time scales. And another one, a minor, direct channel where it quickly depopulates the ππ* state to reach the ground state within tens of fs.^10–13^ In more detail, many (other) theoretical studies have shown that uracil undergoes ultrafast relaxation processes involving several conical intersections (CoIns) to populate the ground state, S_0_, from the electronically excited states on ps or sub-ps time scales.^10,14–20^ Quantum chemical calculations (such as TD-DFT, CASSCF, MRCIS and XMS-CASPT2) indicate that photoexcitation predominantly populates the bright S_2_ state, characterized by a ππ* configuration.^12^ A few tenths of an electronvolt below S_2_ is the dark S_1_ state, which has predominantly nπ* character. Nonradiative decay pathways are available through CoIns between S_2_ and S_1_, as well as between S_1_ and S_0_, facilitating efficient internal conversion. Alternatively, the population can remain trapped in the S_2_ state for several picoseconds, before decaying either directly to the ground state or via the intermediate S_1_ state.^13,14,18,21,22^

Ultrafast time-resolved spectroscopies have pointed out similar time scale for monoexponential decay to the ground state.^22,23^ Also multiexponential decay has been established by ultrafast pump-probe photoionization experiments on isolated nucleic acid bases, with components in the femtosecond,^22,24^ picosecond,^10,23^ and nanosecond^25^ ranges. Several authors have previously proposed that the shortest of these lifetimes, corresponds to decay from the initially excited state S_2_, to the lower S_1_ state.^15,16,24,26^ The observed lifetimes can be significantly impacted by seemingly small changes of molecular structure. For instance, for thymine, which has a very similar structure to that of uracil only differing by one methyl group, the picosecond lifetime component differs by nearly three times, with 6.4 ps for thymine and 2.4 ps for uracil.^18,23^

One technique that has been used to probe nucleobases is X-ray absorption spectroscopy (XAS). XAS comprises a set of advanced techniques which allow for probing the local electronic structure of a molecule, with atomic resolution. Time-resolved X-ray absorption spectroscopy (TR-XAS) extends conventional XAS to the time domain by combining ultrafast pump–probe techniques with X-ray absorption measurements.^27,28^ This made it possible to observe how molecules evolve after photoexcitation, revealing details of nuclear rearrangements^29^ and electronic motion.^30^ In parallel, theoretical studies use simulated spectra to interpret these processes and to examine the role of conical intersections in nonadiabatic dynamics,^31–33^ by identifying changes in electronic-state populations,^20,34^ analyzing nuclear motion,^20,35^ and following wavepacket evolution.^36,37^

In this work, we focus on one specific type of XAS: Near-Edge X-ray Absorption Fine Structure (NEXAFS) spectroscopy, and its time-resolved variant, TR-NEXAFS. NEXAFS targets the near-edge region of an absorption spectrum,^37,38^ complementing other techniques such as time-resolved photoelectron spectroscopy.^10^ NEXAFS is particularly sensitive to local electronic structure, bonding environments, and unoccupied molecular orbitals. This makes it a powerful probe of ultrafast electronic and structural dynamics not only but in particular, for light elements such as C, N, and O, which are the key components of nucleobases. However, the interpretation of such spectra remains challenging without a direct link to the underlying nuclear and electronic dynamics. Here, we provide a direct correlation between non-adiabatic, Trajectory Surface Hopping (TSH) dynamics staying in the context of the mentioned indirect pathway (i.e., relatively long trapping in the S_1_ state) and TR-NEXAFS spectra calculated “on the fly” using a previously developed Density Functional Theory Transition Potential Δ-Kohn-Sham (TP-DFT/ΔKS) approach^39,40^ for the uracil molecule. In fact, it is the transient trapping in S_1_ after excitation to S_2_, *i.e.*, the decisive step in the dominant, indirect relaxation pathway, which is the focus of the present work (see below). By combining the two theoretical approaches just mentioned, we provide a detailed atom- and site-specific, and time-resolved analysis of the early excited-state dynamics of uracil using TR-NEXAFS at the O, N, and C K-edges. Our study treats *all* nuclear degrees of freedom (albeit classically), thus complementing a recent two-dimensional quantum dynamics work by Bäuml *et al.* ^20^

Our paper is organized as follows. In the next chapter, Section 2, we describe computational methods and numerical details used in this work. In Chapter [Sec sec2.4], we characterize (optical absorption) spectra as well as details of the non-adiabatic dynamics in uracil after initial photoexcitation, the latter based on TSH simulations. In Section 3.2, NEXAFS and TR-NEXAFS spectra at the O, N, and C K-edges are presented, which serve mostly as predictions for time-resolved experiments to come, and to demonstrate how electronic and nuclear motion translates into spectroscopic signals. A final Chapter [Sec sec4] summarizes and concludes this work.

## 2 Computational Methods and Details

### 2.1 Ground-State Geometry and Vibrations

First, a ground-state (S_0_) geometry optimization and vibrational frequency calculations by Normal Mode Analysis (NMA) were performed using Density Functional Theory (DFT) at the B3LYP/def2-SVP^41–43^ level of theory. To improve the treatment of noncovalent interactions, Grimme’s D3 dispersion correction with Becke-Johnson damping was employed,^44,45^ as implemented in the ORCA 5.0.4 software package.^46^ The optimized structure is shown in Figure 1b, along with atom numbering used later in this work. The vibrations are needed for Wigner sampling of initial geometries for dynamics and UV/vis spectra (see below).

**Figure 1:**
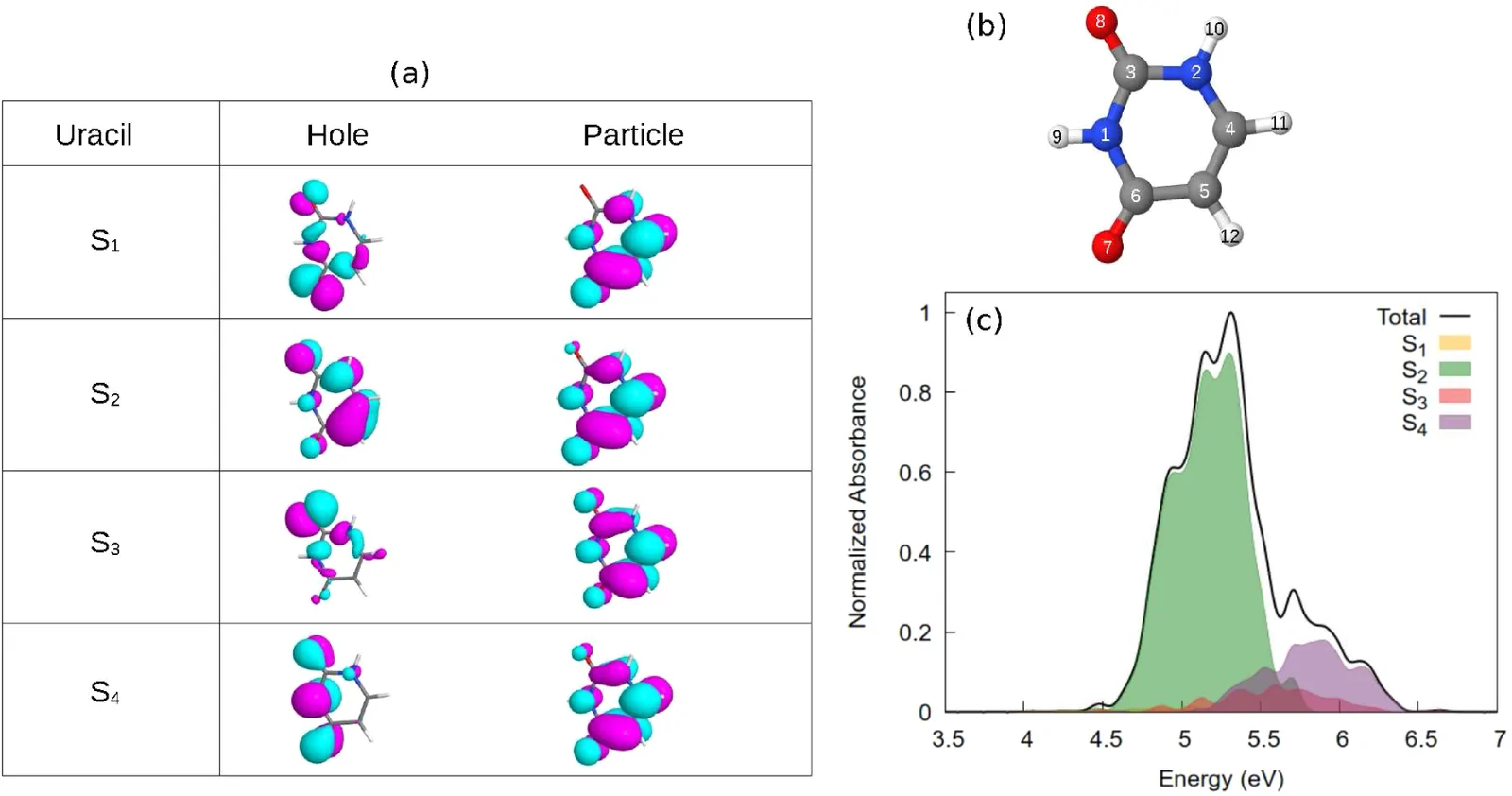
(a) Dominant NTOs for uracil, for S_1_ to S_4_. (b) Optimized geometry of uracil at the B3LYP+D3/def2-SVP+D3 level, with atom numbering indicated. (H: white, N: blue, O: red, C: grey.) (c) Absorption spectra (total and state-resolved) for 400 Wigner-sampled geometries for uracil at TD-B3LYP/def2-SVP level of theory, using eq 1 and S_1_–S_4_. Note that the S_1_ signal (located between ∼4.0 and 5.5 eV) is weak.

### 2.2 Excited States and UV/Vis Absorption Spectrum

First, vertical excitation energies and oscillator strengths for the lowest few singlet excited states were computed, out of the optimized ground-state geometry, using linear-response time-dependent DFT (TD-DFT) at TD-B3LYP/def2-SVP level of theory. These properties were used both to guide the selection of bright initial states for dynamics, and to compute absorption spectra.

An UV/vis absorption spectrum of uracil was simulated to select the photoexcitation window used in dynamics. For this, 400 initial conditions/geometries were generated using a Wigner distribution^47^ based on the harmonic frequencies obtained from the NMA of the optimized ground state. For each of the initial conditions, the lowest four singlet states (S_1_–S_4_) were calculated at the TD-B3LYP/def2-SVP level, and used to determine an (approximate) UV/vis absorption spectrum, as

1 $$I\left(E\right)=K\underset{i,\alpha }{\sum }f_{i,\alpha }\exp\left[-\frac{1}{2\kappa^{2}}{\left(E-E_{i,\alpha }\right)}^{2}\right]$$

Here, *E* = *ℏω* denotes the photon energy, *E*_*i*,*α*_ and *f*_*i*,*α*_ correspond to the calculated excitation energy and oscillator strength, respectively, for the S_0_ → S_*i*_ transition of geometry (“snapshot”) *α* (*i* = 1–4, *α* = 1–400). A Gaussian broadening to the individual transitions was used, with a broadening parameter, *κ* = 0.1 eV, as a default provided in the SHARC interface^48,49^ (with which the dynamics were done). Further, *K* was chosen to normalize the maximal intensity of the broadened spectrum to 1.

### 2.3 Nonadiabatic Trajectory Surface Hopping Dynamics

To investigate the excited-state relaxation dynamics of uracil, nonadiabatic molecular dynamics simulations were performed using Tully’s Fewest Switches Surface Hopping (FSSH) algorithm^50^ as implemented in SHARC 3.0.^48,49^ The quantum chemical backend used throughout the simulations was ORCA 5.0.4, employing the TD-B3LYP/def2-SVP level of theory with tight self-consistent field convergence criteria (as defined in ORCA). As for spectra, the five lowest singlet states (S_0_–S_4_) were included in the simulations. We note that triplet states were not considered in this work. As initial states for dynamics, those excited states, sampled at the geometries corresponding to the Wigner distribution, which had the highest transition dipole moments within an energy window from 4.0–6.0 eV, were selected. The chosen energy window covers the calculated absorption band of uracil (see Figure 1c). A total of 295 initial conditions were selected in this way, most starting in S_2_, which is the first bright (ππ*) state.

The selected initial configurations were propagated for 1 ps with a nuclear integration time step of 0.5 fs. The nuclear motion was propagated using a velocity Verlet integration,^51^ with 25 electronic substeps per nuclear time step to propagate the electronic wavefunction using a local diabatization scheme to facilitate efficient evaluation of nonadiabatic couplings.^52,53^ Upon successful hops between electronic states, nuclear velocities were uniformly rescaled to conserve total energy. The hops were treated with standard hopping probabilities from SHARC. Additionally, an energy-based decoherence correction was included following the approach of Granucci and Persico.^54,55^

Of the initial set of 295 trajectories, 137 were retained for ensemble analysis. Trajectories that violated energy conservation (20 trajectories) exhibited excessively large energy differences in total energy between two consecutive time steps (>0.15 eV, 50 trajectories), showed nonphysical hopping energies (where after a successful hop, the energy changed by more than 1 eV, 35 trajectories) and trajectories that crashed due to convergence problems were discarded (53 trajectories). Analyzed properties are time-resolved state populations, state energies, geometric variables, and, most importantly, NEXAFS spectra.

### 2.4 (Time-Resolved) Near Edge X-ray Absorption Spectra

NEXAFS as well as TR-NEXAFS spectra were simulated, the latter to track the electronic and structural post-excitation evolution of uracil. The method to compute TR-NEXAFS spectra relies on computing pump-probe NEXAFS (PP-NEXAFS) spectra, where a valence excitation (pump) creates an excited state S_*n*_ (*n*  >  0), which is then followed by an X-ray probe leading to site-specific core excitations into an unoccupied orbital. All virtual orbitals are used as final states. The spectra were calculated using the Density Functional Theory Transition Potential ΔKohn-Sham (TP-DFT/ΔKS) approach,^39,40^ wherein a core spin-orbital *ψ*_*i*_ is populated with half an electron (as opposed to one electron) to approximate transitions to final, core-excited states efficiently (this approximation is also called the half-core-hole approximation). Absorption intensities were computed using Fermi’s Golden Rule. Within the method, a ground state NEXAFS spectrum (at *t* = 0) is calculated as^39,40^

2 $$\sigma_{\mathrm{GS}}\left(\omega ,t=0\right)=C\omega \underset{i}{\sum }\underset{f}{\sum }{|\mathrm{e⃗}\langle \psi_{i}|\mathrm{r⃗}|\psi_{f}\rangle |}^{2}\delta \left(\varepsilon_{f}-\varepsilon_{i}-\hbar \omega \right)$$

Here, *e⃗* is the field-polarization vector (averaging over possible polarization directions was done as described in ref 56), *C* a constant. Further, *ψ*_*i*_ is the (half-occupied) initial, KS core orbital *i*, *ψ*_*f*_ is a final (empty, but bound) orbital, and the *ε*_*i*,*f*_ are corresponding energies. Orbitals and energies are obtained from self-consistent TP-DFT. Within TP-DFT/ΔKS, in addition the lowest excitation energy is shifted to align with the energy difference between the first fully core-excited state and the ground state, obtained from a separate ΔKS calculation. Note that initial-state/atom resolved cross sections, *σ*_GS_,*_i_*, can simply be obtained by selecting a certain initial orbital/atom, without summing over *i*. The same way, one can calculate total pump-probe NEXAFS spectra, *σ*_PP_(*ω*, *t*), where (the half-electron) core excitation starts from a valence-excited reference state, S_*n*_ (*n* > 0). Again, a ΔKS shift is applied, but now to align with the energy difference between valence-excited and core-excited states (see refs 39,40 for details). The PP-NEXAFS spectrum *σ*_PP_(*ω*, *t*), and initial-state/atom resolved variants of it, *σ*_PP,*i*_(*ω*, *t*), are here time-dependent, since they arise from a TSH scheme, *i.e.*, the atoms move. This way, TR-NEXAFS spectra are generated “on the fly”.

In practice, we combine TSH with the PSIXAS plugin,^40^ interfaced with the Psi4 electronic structure package.^57^ The B3LYP/def2-SVP level of theory (*i.e.*, the same as for the TSH calculations) was employed for all X-ray absorption simulations. For each selected trajectory, (PP-)­NEXAFS spectra were computed at regular 10 fs intervals from 0 to 1000 fs. Each spectrum was constructed by identifying, from the SHARC/ORCA outputs at time *t*, the trajectory’s current active state, S_*n*_, and geometry, and the corresponding (PP-)­NEXAFS spectrum is calculated. Shown below are *difference spectra* (which are usually reported in experimental studies), computed as

3 $$\Delta \sigma \left(\omega ,t\right)=\left\langle \sigma \left(\omega ,t\right)\right\rangle -\left\langle \sigma_{GS}\left(\omega ,t=0\right)\right\rangle$$

Here, ⟨*σ*(*ω*, *t*)⟩ is an *ensemble-averaged* (PP-)­NEXAFS spectrum at time *t*, calculated as PP-NEXAFS spectrum if the system is in an excited state S_*n*_ (*n* > 0) at time *t*, or as a ground state NEXAFS spectrum when the system is in S_0_ at that time. Further, ⟨*σ*_GS_(*ω*, *t* = 0)⟩ denotes the *ensemble-averaged* GS-NEXAFS spectrum, at *t* = 0. Again, both total and atom/site-resolved spectra can be calculated. Below, the TR-NEXAFS spectra for oxygen, nitrogen, and carbon K-edges were calculated to monitor atom-type and even site-specific changes. A subset of the SHARC trajectories was selected for the TR-NEXAFS analysis. Since the focus of the present work is the early S_2_ → S_1_ internal conversion, mainly trajectories initiated in the S_2_ state were considered, resulting in an ensemble of 120 trajectories for the O 1s and N 1s simulations. For the C 1s calculations, the ensemble was further reduced to 56 trajectories because the larger number of inequivalent carbon atoms substantially increased the computational cost and memory requirements.

All NEXAFS spectra were broadened using a Gaussian function with *κ* = 1.8 eV instead of the delta function in eq 2. The broadening parameter has been previously established in a separate, yet unpublished experimental-theoretical collaborative *gas-phase* TR-NEXAFS study. The same broadening was also used in an earlier theoretical TR-NEXAFS investigation.^58^

## 3 Results and Discussion

### 3.1 Electronic Excitations, Post-Excitation Dynamics

#### 3.1.1 Vertical Excitation Energies and Nature of Excited States

First, vertical excitation energies of uracil out of the ground state were computed using (TD-B3LYP/def2-SVP) at the B3LYP/def2-SVP+D3 geometry. Table 1 lists the first ten singlet excited states, showing their excitation energies, *E*_*i*_, and corresponding oscillator strengths, *f*_*i*_. The S_2_ state exhibits the highest oscillator strength (0.124), of states below 6 eV, identifying it as the most optically accessible transition from the ground state in this energy range. It lies at 5.32 eV, followed by S_4_ at 5.99 eV, which also has a moderate oscillator strength (0.037). The state energies of S_1_ to S_4_ are in excellent agreement with earlier TD-B3LYP/6-31G* calculations.^59^ In that reference, also better basis sets (and other methods) were tested, shifting excitation energies somewhat: At TD-B3LYP/aug-cc-pVTZ level, for example, the energies of S_1_, S_2_, S_3_ and S_4_, are 4.64, 5.11, 5.64, and 5.74 eV, respectively, *i.e.*, red-shifted by up to ∼0.2 eV w.r.t. Table 1. Experimentally, the lowest bright state, S_2_, is at 5.08 eV (in solution) according to ref 60.

**Table 1:** Vertical Excitation Energies and Oscillator Strengths for Uracil Computed Using TD-B3LYP/def2-SVP

Transition	*E*_*i*_ (eV)	Oscillator Strength, *f*_*i*_
S_0_ → S_1_	4.680	0.000
S_0_ → S_2_	5.322	0.124
S_0_ → S_3_	5.818	0.000
S_0_ → S_4_	5.996	0.037
S_0_ → S_5_	6.251	0.000
S_0_ → S_6_	6.659	0.000
S_0_ → S_7_	6.727	0.107
S_0_ → S_8_	6.785	0.000
S_0_ → S_9_	7.363	0.013
S_0_ → S_10_	7.740	0.429

The electronic characters of low-lying excited states S_1_–S_4_ were characterized via natural transition orbital (NTO)^61^ analysis. In Figure 1a, the dominating NTO “hole” and “particle” pairs are shown, for the lowest four vertical excitations. The first excited state (S_1_) predominantly exhibits nπ* character, originating from nonbonding lone pairs on oxygen atoms transitioning to an antibonding π* orbital. The bright S_2_ state is of ππ* character, involving transitions between a delocalized bonding *π*-orbital and an antibonding π* orbital. The third excited state (S_3_) is of nπ* character again and optically dark. The next bright state, S_4_, is of ππ* type and bright. The shape of the hole NTOs of S_1_ and S_2_ are similar to the highest *n* (HOMO–1) and *π* (HOMO) KS orbitals (see ref 59, for example), and the shape of all particle NTOs shown, is similar to the π* (LUMO) orbital.

We also compared the TDDFT-B3LYP vertical excitation energies with previous experimental data and higher-level theoretical values.^9,12,62^ The calculated excitation energies (4.68, 5.32, and 5.82 eV) are in good agreement with the available low-lying experimental values of 4.38, 5.1, and 6.0 eV for S_1_–S_3_ states, respectively.^9,12^ The calculated TDDFT values also compare favorably well with the CASPT2 results, which predicts S_1_ to S_3_ energies in the range of 4.9–6.41 eV and 4.85–6.02 eV depending on the chosen active space, while CASSCF predicts the excitation values in the range of 5.13–7.07 eV and 4.83–7.33 eV (a bit higher, as it lacks the dynamic correlation) depending on the chosen active space.^9^ We note, however, that the S_3_ character is highly sensitive to the chosen active space in case of CASSCF and CASPT2. EOM-CCSD yields somewhat higher excitation energies (5.17, 5.56, and 6.52 eV for S_1_, S_2_, and S_3_, respectively).^62^ Overall, the TDDFT/B3LYP excited states reproduce the relative ordering of the low-lying excited states (S_1_ is nπ*, S_2_ is ππ*) and TD-B3LYP excitation energies are in reasonable agreement with both experiment and higher-level correlated electronic structure methods in the Franck–Condon region, supporting its suitability as the starting point for the present nonadiabatic dynamics simulations.

The features of these electronic transitions are directly reflected in the simulated absorption spectrum for 400 sampled geometries (Figure 1c), which shows a dominant band around 5.3 eV attributed to excitation into S_2_ (green). The second intense peak (less intense than S_2_ though) corresponds to S_4_, consistent with its moderate oscillator strength (violet). These features agree well with previous gas-phase absorption spectra of uracil.^9,13,15,59,62–64^

#### 3.1.2 Post-Excitation Dynamics by Trajectory Surface Hopping

To simulate the ultrafast dynamics following photoexcitation, we initiated the 295 trajectories selected from the Wigner-sampling as described above, by including the S_1_–S_4_ excited states. In the TSH dynamics, done by SHARC, also the S_0_ ground state was included. As said earlier, most trajectories started in state S_2_, namely 234 (∼79.3%), while 14 (∼4.7%) started from S_3_, and 47 (∼15.9%) from S_4_. Out of these, 137 trajectories were retained after applying filters for energy conservation and unrealistic fragmentations (see the Methods section).

Figure 2a shows the evolution of electronic state populations over time. It is seen that the initially dominant S_2_ population is quickly transferred to the first excited state, S_1_. Fitting the rise in S_1_ (nπ*) population to a single-exponential function (1 – exp­(−*t*/*τ*)), yields a characteristic time constant of approximately 140 fs. Mechanistically, after excitation to the bright ππ* (S_2_) state, the system undergoes rapid internal conversion to the dark nπ* (S_1_) state via a well-characterized S_2_/S_1_ conical intersection as mentioned earlier. We also note that for a larger ensemble of 186 trajectories (including all trajectories that reached 1 ps, some of which are not included in the 137-trajectory ensemble based on the energy-related criteria described in the last paragraph of Section 2.3), the S_1_ time constant is very similar, 141 fs. Furthermore, for the smaller samples of 120 and 56 trajectories used for TR-NEXAFS calculations, the S_1_ time constants are 144 and 127 fs, respectively.

**Figure 2:**
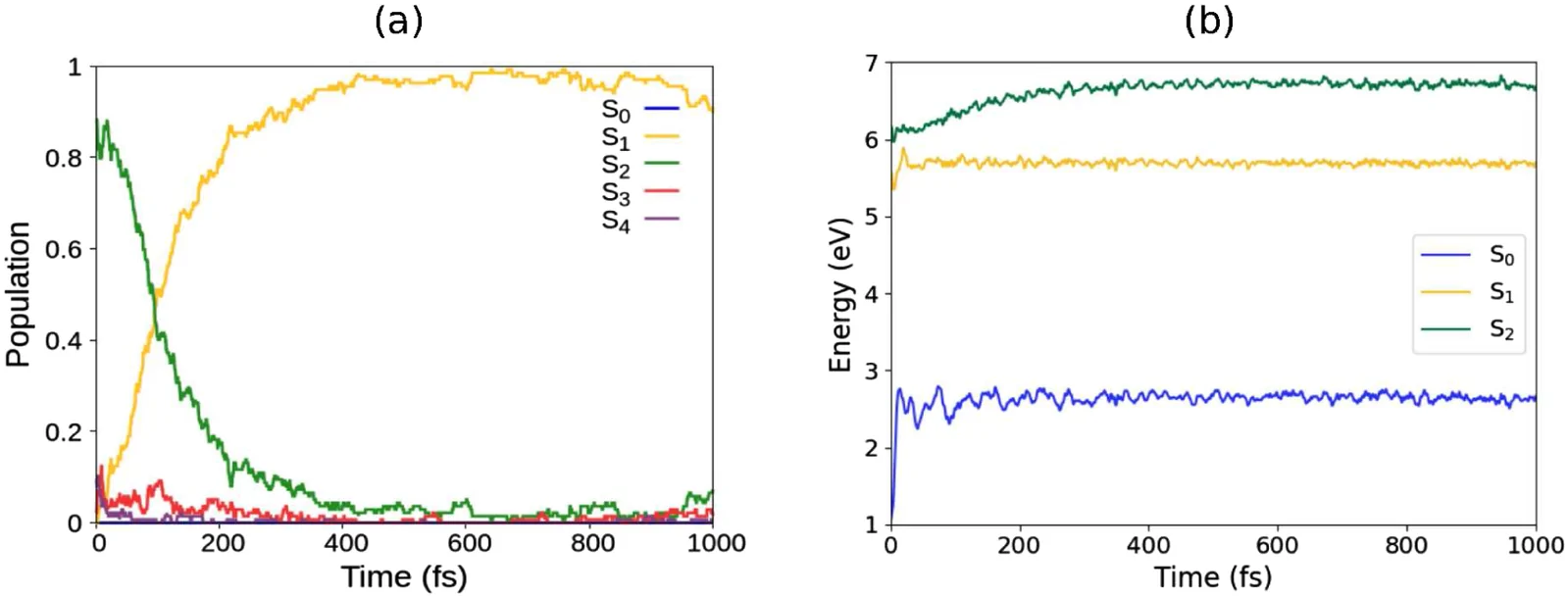
(a) Ensemble-averaged electronic state populations of states S_0_–S_4_ for the uracil molecule as a function of time. (b) Ensemble-averaged energies of states S_0_–S_2_ as a function of time.

No significant population transfer to S_0_ is observed within our simulation window of 1 ps. The lack of ground-state recovery, together with the low populations in S_3_ and S_4_ (see below), suggest that the system becomes transiently trapped on the S_1_ surface. The absence of ground state (S_0_) relaxation can partly be explained by the electronic structure of uracil, where the S_1_ state is known to host a local minimum that is only weakly coupled to the S_1_/S_0_ conical intersection, thus functioning as a transient energy trap.^9,12^ The trapping of S_1_ is also consistent with a persistent energetic gap between the S_1_ and S_0_ surfaces according to our simulations (*cf.* Figure 2b), which prevents relaxation to the ground state. We note that the potential energies shown in Figure 2b represent the dynamical evolution rather than potential energy scans.

We note that the S_1_ trapping found here could also have methodological causes (TD-DFT underestimates S_1_ → S_0_ transitions, we have not included spin-orbit coupling to triplet states, and the propagation time is comparatively short). Furthermore, the absence of S_1_ → S_0_ hops is also likely influenced by the use of TD-DFT, a single-reference method that is generally less reliable in accurately describing regions near conical intersections, notably those involving S_0_. We also note that no S_0_/S_1_ energy gap criterion was used in our simulations to enforce transitions to S_0_. Also, the inclusion of triplet state is expected to lead to S_1_ depopulation via intersystem crossing,^10^ thus reducing the trapping effect. On the experimental side, it has been shown that uracil and related molecules can exhibit delayed fluorescence (or phosphorescence), indicating that the lowest nπ* state is indeed transiently trapped.^14,65^ Further, Ghafur *et al.* ^21^ reported biexponential decay dynamics with a fast sub-100 fs component followed by a much slower relaxation to S_0_ on the picosecond time scale of around 3 ps. Recently, Faccialà *et al.* ^10^ reported ultrafast S_2_ → S_1_ internal conversion within 17 ± 4 fs and showed that the deactivation pathway moves through an intersystem crossing, with S_1_ → T_1_/T_2_ conversion taking place in 1.6 ± 0.4 ps. They also reported on a minor pathway proceeding via ultrafast (tens of fs) ππ* → S_0_ internal conversion. On the theory side, some studies have reported S_1_ → S_0_ internal conversion in uracil. But even there, in multireference simulations, only a small fraction of trajectories exhibited relaxation to the ground state.^12^ Also Richter *et al.* ^9^ have shown that population can transfer from the S_1_ (^1^ nπ*) state to the energetically accessible T_2_ (^3^ ππ*) state, followed by rapid internal conversion to T_1_ before returning to S_0_ with decay time of 2–3 ps. Semiempirical surface hopping simulations suggest that ground-state recovery is delayed, with approximately 90% of the population returning to S_0_ only after about 1.3 ps,^64^ *i.e.*, on somewhat longer time scales. Consequently, while the present simulations only emphasize the indirect but dominant, relaxation channel, they remain consistent with experimental evidence for transient population trapping in the nπ* state and provide insight into the spectroscopic signatures associated with this pathway (presented in Section 3.2). Since our focus here is on this indirect relaxation pathway involving transient population of the lowest nπ* state, the use of TD-DFT is justified.

In Figure 2a, also populations of states S_3_ and S_4_ are shown, which were selected with small probability as initial states (see above). As a consequence, we note that both S_3_ and S_4_ exhibit only transient population following excitation. A small rise in population of S_3_ is observed within the first 50–100 fs due to nonadiabatic transitions from state S_4_ to S_3_. However, this population increase is short-lived, and both states quickly depopulate, predominantly funneling into S_1_ (*i.e.*, S_3_ → S_2_ → S_1_). This is consistent with the expected ultrafast internal conversion dynamics in pyrimidine bases, where the excited-state manifold is densely packed and internal conversion is efficient.^9,12,14,15^

#### 3.1.3 Geometrical Changes during Nonadiabatic Transitions

To elucidate the structural response of uracil following photoexcitation, we examined key geometrical parameters during the early stages of nonadiabatic dynamics. In particular, we focused on changes in carbonyl and ring-associated bond lengths to assess how nuclear motion contributes to internal conversion.

Figure 3a and b show the time evolution of the C6–O7 and C3–O8 carbonyl bond lengths for the 1 ps simulation interval, with atom numbering given in Figure 1b. Both bond lengths oscillate on the time scale of CO vibrations (of ∼20 fs for a vibration with ∼1700 cm^–1^, see also Figure 4). Then they gradually elongate (C6–O7) or shorten (C3–O8), on the time scale of a few hundred fs, which is the time scale of the S_2_ → S_1_ transition. This dynamic redistribution suggests a rapid modulation of electron density within the conjugated *π*-system, consistent with a transition between delocalized ππ* and more localized nπ* states. We also find substantial out-of-plane motion of the O7 atom, previously characterized in multireference studies as the defining feature of the out-of-plane-O intersection,^13^ which is reflected in altered C–O bond geometries in our trajectories.

**Figure 3:**
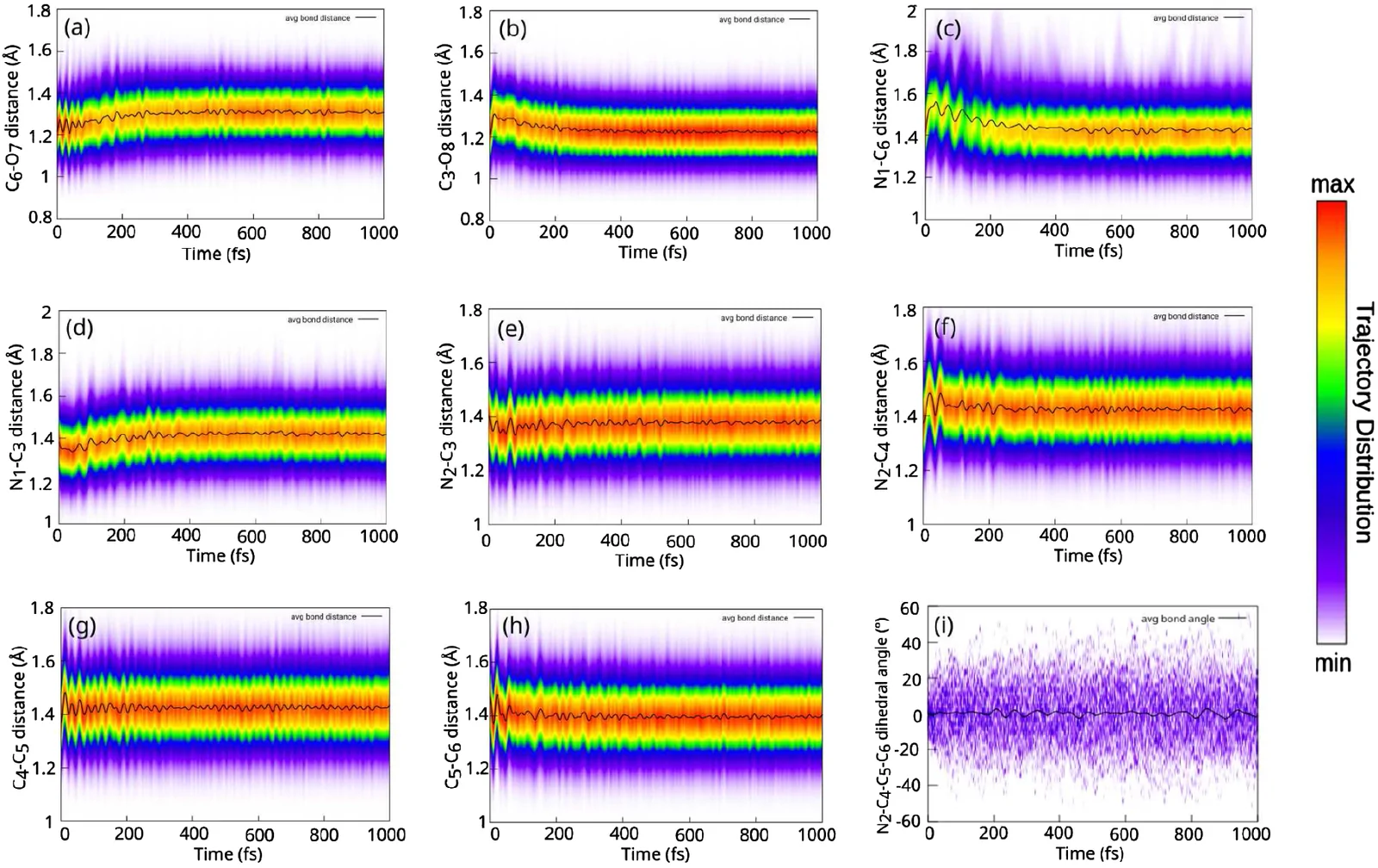
Ensemble-averaged bond lengths and angles in uracil after photoexcitation. (a) C6–O7 bond length; (b) C3–O8 bond length; (c) N1–C6 bond length; (d) N1–C3 bond length; (e) N2–C3 bond length; (f) N2–C4 bond length; (g) C4–C5 bond length; (h) C5–C6 bond length; (i) ensemble-averaged N2–C4–C5–C6 dihedral angle. The colored patterns give the probability distributions and the solid black lines are the averaged values over all analyzed trajectories. Atom numbering is according to Figure 1b.

**Figure 4:**
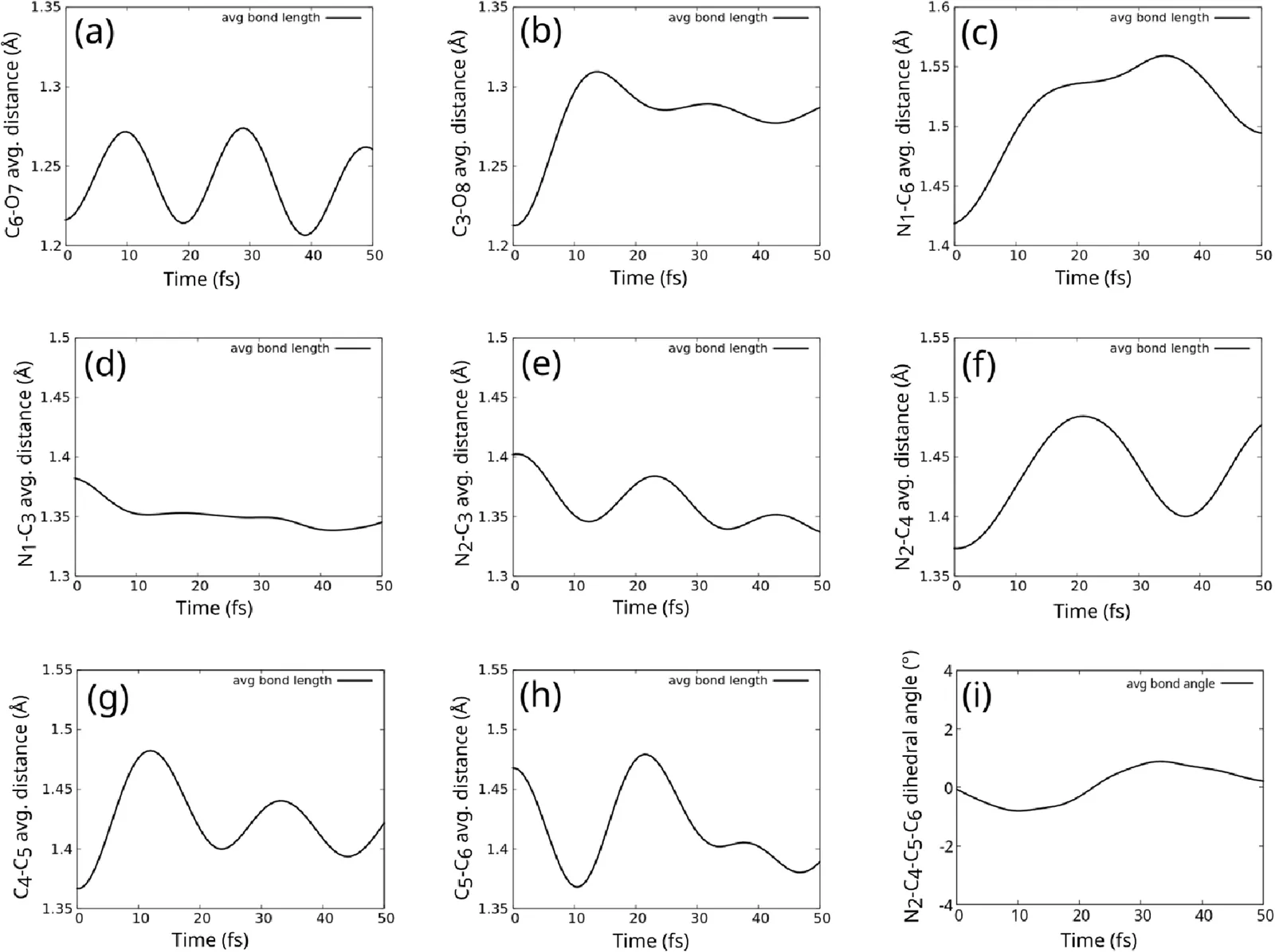
Averaged bond lengths and angles (averaged over all trajectories) in uracil after photoexcitation upto 50 fs. (a) C6–O7 bond length; (b) C3–O8 bond length; (c) N1–C6 bond length; (d) N1–C3 bond length; (e) N2–C3 bond length; (f) N2–C4 bond length; (g) C4–C5 bond length; (h) C5–C6 bond length; (i) ensemble-averaged N2–C4–C5–C6 dihedral angle. Atom numbering is according to Figure 1b.

Beyond the carbonyl groups, notable changes are observed in N1–C6 and N1–C3 bonds (Figure 3c,d). These changes are known to occur near S_2_/S_1_ hopping geometries,^12^ and are structurally aligned with puckering and torsional displacements which drive access to conical intersections.^12,13^ In contrast, the N2–C3 and N2–C4 bonds remain relatively unaffected over time, as shown in Figure 3e,f.

The C4–C5 double bond, part of the ethylenic moiety implicated in one known S_1_/S_0_ CoIn pathway,^9,12,15^ exhibits pronounced damped oscillatory behavior during the first ∼140 fs (see Figure 3g).

In general, the observed geometrical changes are a result of electronic transitions and post-excitation nuclear motion. For instance, after sudden initial S_0_ → S_2_ excitation, the ground-state C4–C5 bond length is too long in the excited state, because a *π* (C4–C5 bonding) → π* (C4–C5 antibonding) transition took place, *cf.* Figure 1. The molecule starts to adapt to the new electronic structure, visible by an elongation of the C4–C5 bond length within the first 10 fs or so, corresponding to about half of the vibrational period of the C–C stretch mode (also ∼20 fs). Then it undergoes the mentioned, damped oscillation (black line in panel­(g)), toward an averaged C4–C5 bond length of slightly above 1.4 Å, which is half-way between a C–C double (∼1.34 Å) and C–C single-bond length (∼1.54 Å). Similarly to C4–C5, also the C–O bond lengths C3–O8 and C6–O7 show a rapid expansion after initial S_0_ → S_2_ excitation. This is in agreement with experimental findings based on O 1s X-ray photoelectron spectra in ref 10. While the C4–C5 bond length does not change a lot during the S_2_ → S_1_ transitions around ∼140 fs, others, like the C3–O8 and C6–O7 bond lengths mentioned above show a clear overall trend during this transition. The precise nature of these trends, however, cannot be easily deduced from the shape of participating NTOs alone, in particular not from those evaluated at the ground state geometry given in Figure 1.

Overall, however, the structural dynamics observed in our TD-DFT based surface hopping simulations are consistent with motion toward well-characterized S_2_/S_1_ intersection seams involving puckering, bond elongation, and lone pair reorganization.^9,10,12,13,15,64^ We note that our time scale for the S_2_ → S_1_ transition ∼140 fs, is somewhat longer than the experimental one found in ref 10, of 17 ± 4 fs. Interestingly, the same 17 fs were inferred from EUV-TRPES experiments.^66^ However, SCS-ADC(2) surface hopping simulations used in ref 10. yield a ππ* decay time of ∼160 fs,^10,67^ which is close to our TD-DFT result. We note though that the computational time scale of S_2_ → S_1_ internal conversion is very sensitive to the electronic structure method used for nonadiabatic dynamics, with CASSCF predicting 575.5 fs and XMS-CASPT2 predicting only 12.5 fs.^12,68^ Also, in our work we find no evidence for direct ππ* → S_0_ transitions, which was an observed minority channel according to ref 10. Despite the difference between the experimental and TD-DFT lifetimes, we expect the trends observed in the TR-NEXAFS spectra reported in the next section remain valid, as they track a transition between different electronic states, only with changes occurring faster in the experimental system.

### 3.2 Stationary and Time-Resolved NEXAFS Spectra

To directly correlate the ultrafast excited-state dynamics of uracil with time-resolved electronic and structural changes, we computed TR-NEXAFS spectra at the O 1s, N 1s, and C 1s edges. First, the stationary absorption characteristics of the ground state (GS), as well as idealized PP-NEXAFS spectra when S_1_ or S_2_ states are assumed to have been completely pre-excited initially (no admixture of the GS), all at the optimized GS geometry, are presented. Then TR-NEXAFS signals will be computed, using geometries and state populations from the TSH trajectories. All NEXAFS spectra were calculated using the TP-DFT/ΔKS approach as described earlier.

#### 3.2.1 O 1s Stationary and TR-NEXAFS Spectra

Let us start with the stationary ground state (GS) O 1s NEXAFS spectrum. The broadened O 1s absorption spectrum in the ground state, shown as blue line in Figure 5a and denoted there as “B3LYP-GS-O1s”, is characterized by the onset of intensity above ∼532 eV, with prominent features extending from 534–537 eV and from 539–543 eV. These transitions originate from excitations of the oxygen 1s core electron(s) into the lowest unoccupied orbitals of uracil. In uracil, there are two O atoms, O7 and O8, and the shoulder at 534 eV, is due to the excitation from the O7 1s orbital, to the lowest π* orbital (the LUMO), while the intensity around the maximum of the peak is dominated by the transition of O8 1s, to the same final orbital. More precisely, the two lowest-energy transitions in the O 1s absorption peak around 534–537 eV, are found at 533.9 eV (O7) and 535.2 eV (O8), and are shown (with intensities) as blue sticks underlying the broadened spectrum. (Note that also other transitions contribute to the main peak.) Experimentally, the gas phase GS O 1s NEXAFS spectrum of uracil was determined in ref 69. The spectrum has a similar overall shape as here, with a higher resolution than chosen here. In experiment, two lowest-energy peaks called “A” and “B” are found, at 531.4 and 532.4 eV, respectively. Obviously, our theoretical spectrum is blue-shifted by about 2–3 eV, and the peak splitting somewhat overestimated by 0.3 eV. Note that in ref 69, also theoretical O 1s GS NEXAFS spectra were determined, using the more accurate ADC(2)/6-31+G method. This method gives the lowest two peaks with the same assignment as here, appearing at 533.5 and 534.5 eV, respectively, after an overall, empirical shift of −2.15 eV was made. In our work, we do not make such empirical shifts, since we are mostly interested in changes of spectra during dynamics (see below).

**Figure 5:**
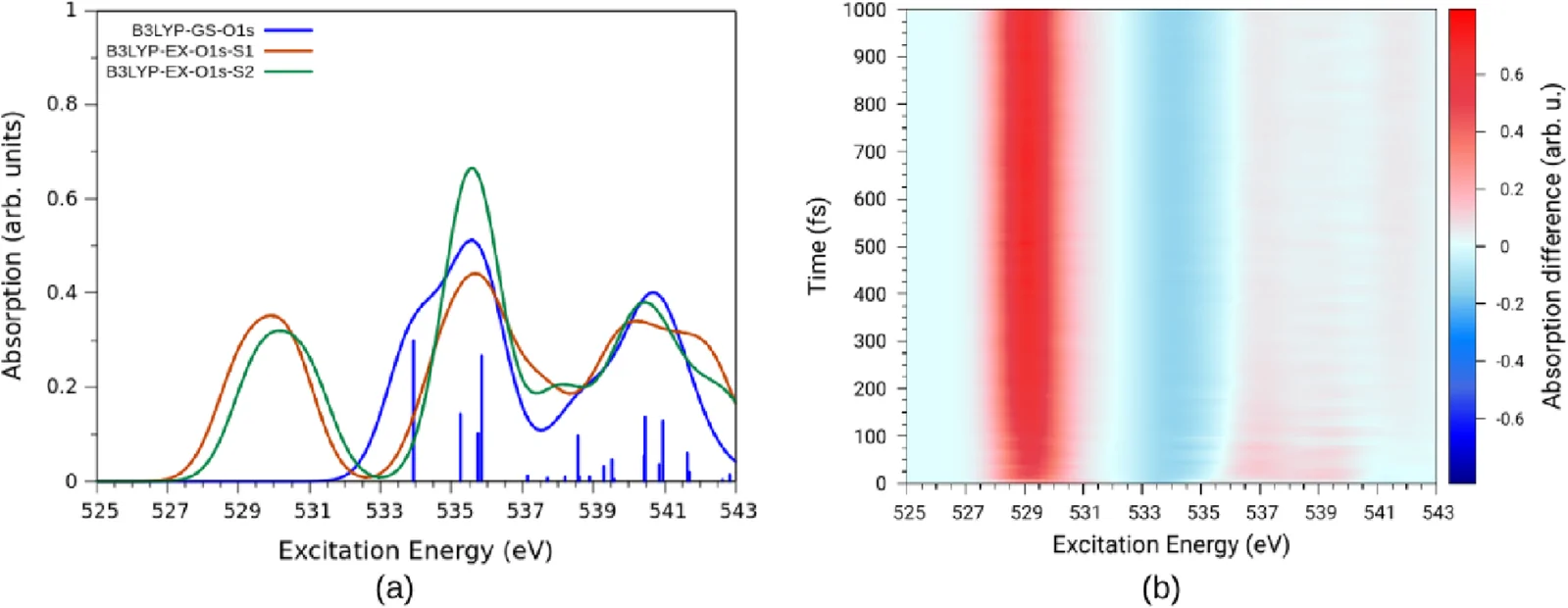
(a) Broadened O 1s GS-NEXAFS spectrum with underlying stick spectrum, both in blue, and PP-NEXAFS spectra for the S_1_ (red) and S_2_ states (green) of uracil. (b) Time-resolved O 1s NEXAFS spectrum (TR-NEXAFS) following photoexcitation.

Upon valence excitation, in the first excited state, S_1_ (nπ*), an electron is promoted from a nonbonding *n* orbital, to the previously empty π* orbital, giving rise to a modified NEXAFS spectrum. The *n*-orbital now being partially empty, allows O 1s → *n* transitions to happen, which leads to an intense lower-energy peak with maximum intensity at 529–530 eV in the PP-NEXAFS spectrum (red line in Figure 5a, labeled “B3LYP-EX-O1s-S1”). The higher-energy peak corresponding to the O 1s → π* transitions is also affected, since π* is now partially filled. Similarly, for the S_2_ PP-NEXAFS spectrum (green line in Figure 5a, labeled “B3LYP-EX-O1s-S2”), the pre-excitation causes a *π* → π* transition, leading to a hole in the *π*-orbital, which, upon probe excitation, causes another low-energy satellite at 530–531 eV in the NEXAFS spectrum due to O 1s → π* transitions. To the best of our knowledge, no PP-NEXAFS spectra of gas-phase uracil have been measured so far.

These features are clearly reflected in the (atom- and trajectory-averaged) oxygen K-edge TR-NEXAFS difference spectrum (Figure 5b), calculated via eq 3. Recall that in the photodynamics, ∼79% of trajectories start in the S_2_ state. As a consequence, we see a strong positive absorption band centered around 529 eV, corresponding to the lowest-energy feature in the PP-NEXAFS (S_2_) spectrum in Figure 5a, green line. We also see a weaker, transient feature near 537 eV. At early times (0–20 fs), the 529 eV band appears sharp and intense, but it rapidly broadens and stabilizes by approximately 140 fs. This evolution reflects the effects of vibronic coupling and nuclear ensemble dispersion, as the molecule (trajectories) explore(s) a range of nuclear geometries on the excited-state potential energy surfaces. This behavior is in line with prior time-resolved XAS simulations, where the evolving spectral signatures were attributed to population-weighted contributions from the S_2_ and S_1_ states and their associated nuclear geometries.^20^

The higher-energy absorption feature initially centered around 536 eV (Figure 5b), displays a distinct temporal evolution that is highly diagnostic of excited-state relaxation pathways. At early times (within the first 20 fs), the absorption begins near 536 eV and progressively shifts toward higher energies, peaking around 537 eV before decaying in intensity after 100 fs. This gradual blue-shift indicates a dynamic change in the local electronic environment of the carbonyl oxygen atoms and is consistent with the onset of nonadiabatic transitions from the initially excited S_2_ (ππ*) state to the lower-lying S_1_ (nπ*) state. The precise nature of blue-shift and fading of this signal is difficult to analyze, due to the underlying nuclear dynamics. However, the intensity loss and blue-shift at longer times are already consistent with the lower intensity/blue-shift of this peak in the stationary PP-NEXAFS (S_1_) spectrum, compared to the corresponding peak in the PP-NEXAFS (S_2_) spectrum.

A prominent negative signal is also observed between 532–536 eV, most intense at 534 eV. This feature corresponds to a ground-state bleach after photoexcitation. At time zero, the bleach spans from 533–535 eV, but over the first 100–150 fs, it shifts toward 536 eV (its right side), forming a curved temporal profile before stabilizing.

We also performed an atom-resolved analysis of the O 1s TR-NEXAFS spectrum (Figure 6a,b). A pronounced asymmetry is observed between the two oxygen atoms, O_7_ and O_8_, reflecting their distinct electronic roles during the excited-state dynamics. O_7_, bonded to C_6_ exhibits dominant and dynamically evolving spectral features, while O_8_, bonded to C_3_ between the two nitrogen atoms (see Figure 1b), shows a relatively weaker response but also dynamically evolving.

**Figure 6:**
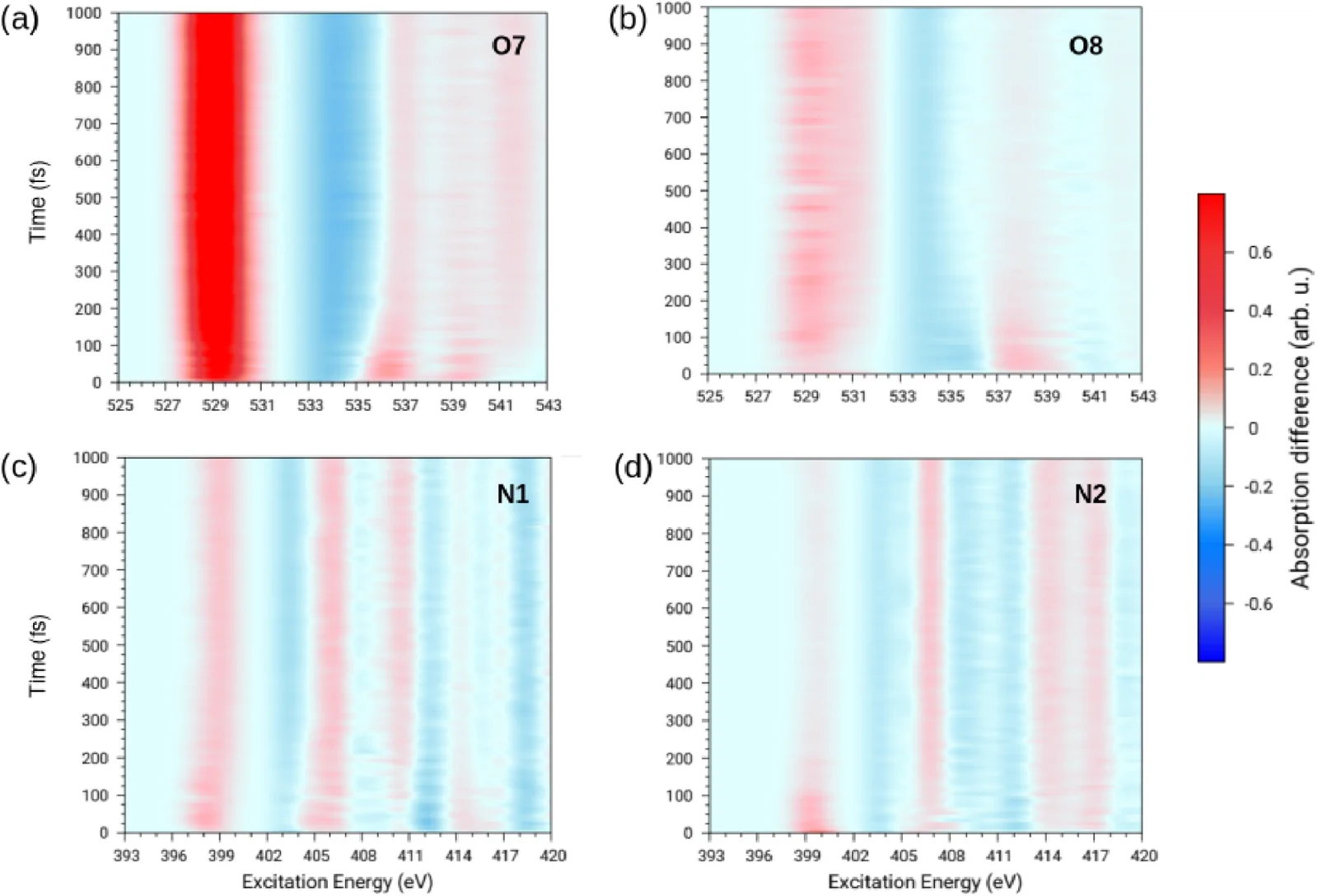
Atom-resolved TR-NEXAFS of the uracil molecule. (a) and (b) represent the O7 and O8 atom-resolved TR-NEXAFS, (c) and (d) represent the N1 and N2 atom-resolved TR-NEXAFS. See Figure 1b for atom numbering.

The O7-resolved spectrum displays two key positive band features: a strong, stationary absorption near 529 eV and a dynamic peak that shifts from approximately 535 eV to 537 eV within the first ∼140 fs. A ground-state bleach is also present, initially peaking at 534 eV and shifting its right side to 536 eV in parallel with the 535 to 537 eV positive feature. This correlated blue-shift closely tracks the lengthening of the C6–O7 bond observed in the excited-state dynamics (Figure 3a). We note that here, and in what follows, we compare changes in *ensemble-averaged* TR-NEXAFS with evolution of *ensemble-averaged* geometrical parameters.

In contrast, for the O8-resolved spectrum, a weak and relatively stationary peak at 529 eV is concurrent with the shortening of the C3–O8 bond (Figure 3b) The next features of the O8 spectrum are the ground state bleach at around 534 eV and a positive band at 538 eV. The border between these features experiences a red-shift with time in around 150 fs, and the positive band also looses its intensity within this time. As a result, the O8 site contributes less to the evolving absorption spectrum, due to diminished oscillator strength of the S_1_ excited state.

Overall, our O 1s NEXAFS spectra are associated with ultrafast S_2_ → S_1_ internal conversion, consistent with the surface hopping results showing system relaxation within the 140 fs timeframe. The redistribution of valence electron density is directly reflected in the evolving spectral features of a nonadiabatic relaxation pathway from the more delocalized ππ* excitation to the more localized nπ* state in uracil.

#### 3.2.2 N 1s Stationary and TR-NEXAFS Spectra

The calculated N 1s GS-NEXAFS and stationary S_1_ and S_2_ PP-NEXAFS spectra of uracil are shown in Figure 7a. For the GS spectrum, two main bands are observed in the energy range shown, a highly structured lower-energy one extending from ∼403–413 eV, and a higher one from ∼415 to above 420 eV. More precisely, the two lowest-energy transitions from N 1s to the LUMO (π*), are at 403.7 eV for N1, and at 404.0 eV for N2, respectively, *i.e.*, both are under the shoulder around 404 eV in the figure (see also the underlying stick spectrum). Experimentally, this shoulder is a (better resolved) signal called “A” in ref 69, with its maximum at 401.4 eV. Calculations in ref 69 on the ADC(2)­s/6-311+G* level show that the two lowest-energy N 1s NEXAFS signals dominate the “A” peak, and are found, after an empirical red-shift by −3.80 eV, at 400.5 eV (N1 1s → π* (LUMO) transition) and 400.8 eV (N2 1s → π* (LUMO) transition), respectively. Hence, our TP-DFT/ΔKS calculations are blue-shifted again by ∼2–3 eV w.r.t. experiment; the assignment and splitting of the two lowest peaks are consistent with the ADC calculations in ref 69.

**Figure 7:**
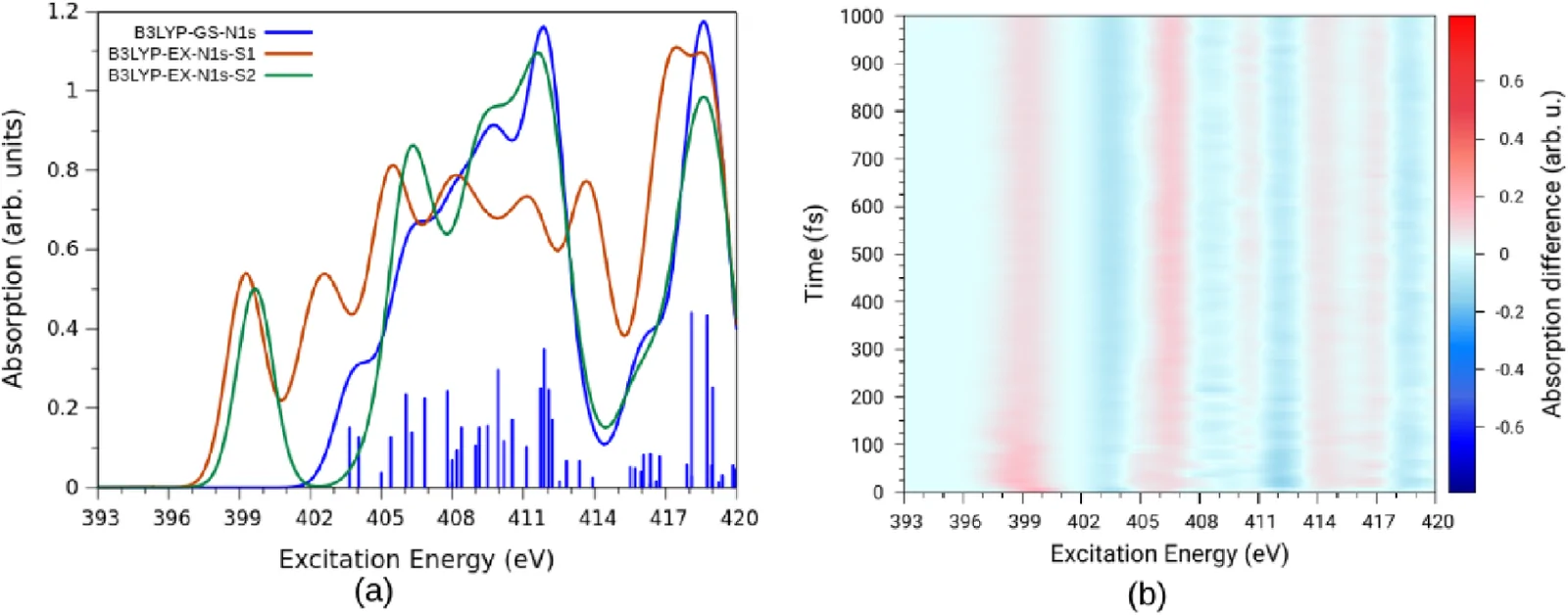
(a) Broadened N 1s GS-NEXAFS spectrum (with underlying stick spectrum) and PP-NEXAFS spectra for the S_1_ and S_2_ states of uracil. (b) Time-resolved N 1s NEXAFS spectrum (TR-NEXAFS) following photoexcitation.

According to Figure 7, for the PP-NEXAFS spectra strong new peaks are observed at 399 eV (S_1_, red) and close to 400 eV (S_2_, green), respectively, due to the holes in previously occupied *n* and *π* orbitals, respectively, for the valence-excited states – in full analogy with the O 1s PP-NEXAFS spectra.

Analyzing the time-resolved N K-edge difference spectrum TR-NEXAFS in Figure 7b, we first note two positive absorption bands (red areas) near 399 and 406 eV. The lower-energy band at 399 eV shows a gradual decrease in intensity over the first ∼150 fs, accompanied by a small blue-shift (by less than 1 eV). The most prominent low-energy ground state bleach (blue) is found near 403 eV, where the GS NEXAFS spectrum showed a shoulder (the lowest-energy peak “A” in ref 69). In the stationary S_1_ PP-NEXAFS spectrum, a distinct feature was also observed at 402 eV; however, no corresponding signal is present in the TR-NEXAFS spectrum, despite one might expect such a feature emerging when a S_2_ → S_1_ transition took place. Apparently, the lack of such a feature (associated with S_1_) in the TR-NEXAFS spectrum is caused by a dynamical effect. In addition to the spectral intensity in the low-energy region, a new absorption feature emerges near 410 eV at later times, around 100 fs. At similar time a positive features disappears around 416 eV. Such behavior could be associated with changes in electronic character or molecular geometry during excited-state relaxation.

The atom-resolved N 1s TR-NEXAFS spectra were computed (Figure 6c,d). For N2-resolved spectra, a main absorption feature near 399 eV gradually decreases in intensity after approximately 100 fs, consistent with population transfer from S_2_ to S_1_ seen in TSH simulations. The average N2–C3 and N2–C4 bond lengths do not exhibit substantial net change over time (see Figure 3e,f), short-time oscillations are observed within the first ∼150 fs. Concurrently the absorption feature near 407 eV demonstrates some oscillations, and remains relatively static in both energy and intensity after ∼150 fs. The 410 eV positive feature, which was seen in case of overall N 1s TR-NEXAFS spectrum (Figure 7), is absent in the N2-resolved spectrum (Figure 6c). This indicates that the appearance of 410 eV feature is not associated with N2.

In contrast, N1, the N atom sandwiched between the two C=O groups, exhibits more pronounced temporal changes. The higher-energy absorption band, initially spanning 414–417 eV, gradually decreases in intensity. Subsequently, a new feature at approximately 410 eV appears within the first ∼150 fs. Meanwhile, the lower-energy feature near 398 eV shows a very slight blue-shift toward 400 eV. The spectral appearance at ∼410 eV is attributed to internal conversion from the bright S_2_ to the dark S_1_ state, accompanied by nuclear motions localized around the N1 site. These spectral features likely arise from orbital reorganization and reduced oscillator strength linked to local bonding changes, such as the shortening of the N1–C6 bond and elongation of N1–C3 (see Figure 3c,d).

Overall, also the N 1s NEXAFS spectra reflect the S_2_ → S_1_ nonadiabatic transition on a ∼140 fs time scale, after which only weak residual spectral features coming from S_1_ remain, showing that internal conversion to the ground state (S_0_) occurs on a longer time scale than covered in the present simulations. Further, a clear asymmetry between the N atoms is seen, similar to the one for the two O atoms, highlighting the sensitivity of TR-NEXAFS to both local bond reorganization and nonlocal electronic effects.

#### 3.2.3 C 1s Stationary and TR-NEXAFS Spectra

Finally, the C 1s NEXAFS spectra of uracil were studied. The static GS-NEXAFS and S_1_, S_2_–PP-NEXAFS C 1s spectra are presented in Figure 8a. For the C 1s GS-NEXAFS spectrum, absorption starts at ∼287 eV with continuous increase in intensity until 307 eV. Closer inspection shows that under the low-energy peak centered around 288 eV, four dominating peaks are found, corresponding to excitations from the four C 1s orbitals into the LUMO, at 287.3 eV (for C5), 288.6 eV (for C4), 290.7 eV (C6), and 292.5 eV (C3). The experimental C 1s NEXAFS spectrum of uracil finds four well-resolved lowest-energy peaks “A” to “D” in ref 69, at 284.8, 286.1, 288.0, and 289.5 eV, respectively. According to ADC(2)/6-31+G calculations, these peaks are dominated by the C 1s → π* (LUMO) transitions, located (after an empirical −2.46 eV shift), at 284.4 eV (C5), 286.1 eV (C4), 288.0 eV (C6), and 289.5 eV (C3), respectively. That is, w.r.t. experiment we have a blue-shift again (of ∼2–3 eV), while the peak splittings are very good in this case. ADC2 gives similar results^69^ when no shift is made, and the assignment of dominant peaks contributing to A-D is the same as here.

**Figure 8:**
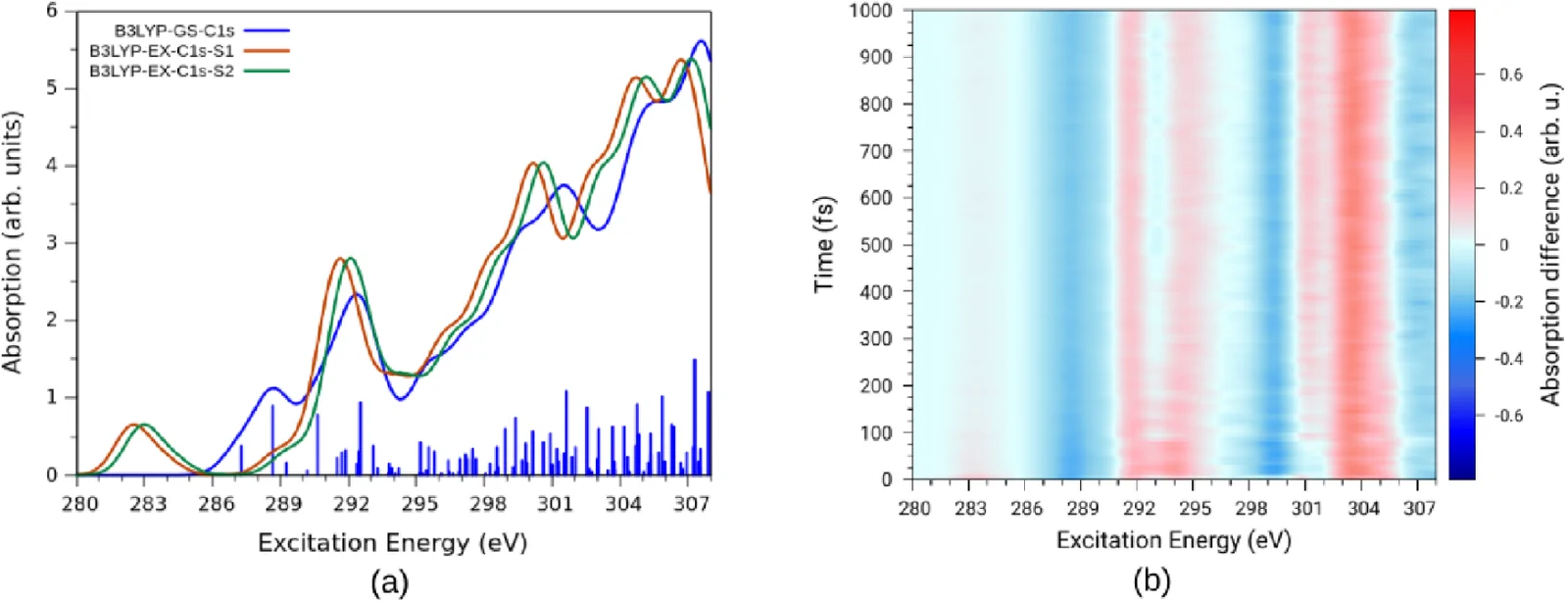
(a) Broadened C 1s GS-NEXAFS spectrum (with underlying stick spectrum) and PP-NEXAFS spectra for the S_1_ and S_2_ states of uracil. (b) Time-resolved C 1s NEXAFS spectrum (TR-NEXAFS) following photoexcitation.

Concerning the PP-NEXAFS spectra, we see from Figure 8a for both S_1_ and S_2_ new absorption features at 282–284 eV. Again, the additional signals reflect transitions into the hole orbitals created by the initial excitation, where the S_1_ peak at 282 eV is due to the hole generated in the *n*-orbital and the S_2_ peak at 283 eV due to the hole in the *π*-orbital. Further, the ground state signal around 288 eV, has clearly lost intensity in the PP-NEXAFS spectra, which would probably contribute to a ground state bleach in the C 1s TR-NEXAFS spectra. Finally, at the higher-energy region around 298–305 eV, S_1_ and S_2_ peaks are shifted slightly relative to GS, which may manifest in TR-NEXAFS spectra as well.

The carbon K-edge TR-NEXAFS difference spectrum (Figure 8b) consists of three main positive absorption bands centered near 283, 293, and 304 eV, each showing distinct temporal evolution. The lowest-energy ground state bleach (blue areas) is observed near 288 eV, as expected. The earliest spectral feature at 283 eV, undergoes a rapid decay within the first 15–20 fs. This transient signal loss is attributed to immediate structural deformation in the S_2_ excited-state, particularly involving C–C double bond elongation and initial out-of-plane distortions of the pyrimidine ring after photoexcitation. Our surface hopping simulations confirm that strong out-of-plane distortions (strong change in dihedral angles) and bond-length modulations (see Figure 3g–i and Figure 4g–i) occur within this timeframe, especially at C4–C5 and adjacent atoms, disrupting *π*-conjugation and thereby altering the character of high-lying occupied orbitals (participating in hole-refilling). These geometric perturbations presumably reduce the overlap of core to valence transitions that initially (*i.e.*, after initial excitation mainly into S_2_), contribute to the 283 eV band, leading to its ultrafast disappearance.

The second decaying, positive feature of interest, centered at 293 eV, decreases more slowly and is largely suppressed by around 100 fs. This evolution coincides with the internal conversion from the bright S_2_ (ππ*) state to the dark S_1_ (nπ*) state, as revealed by the surface hopping population dynamics (Figure 2). Concurrently, a new absorption feature emerges around 301 eV, marking a distinct spectral signature of S_1_ population. Although both states (S_1_ and S_2_) have comparable static intensities at this energy, the dynamic buildup of this feature correlates with the rise of S_1_ population, suggesting that structural and electronic relaxation in the nπ* state enhances the relevant core to valence transitions. In particular, reorganization of the low-lying unoccupied orbitals influenced by lone-pair participation on heteroatoms and altered *π*-conjugation modifies their energy levels and spatial overlap with carbon 1s orbitals, thereby enabling transitions not prominent at earlier times. The spectral contrast between the 293 eV decay and the 301 eV rise is consistent with electronic state conversion. We also note that the C 1s TR-NEXAFS spectral features were found to be preserved across different ensemble sizes, as shown by comparisons using 20 and 40 trajectories (see Supporting Information, Figure S1).

In addition to electronic effects, the C 1s TR-NEXAFS spectra reveal a strong sensitivity to site-specific structural reorganization. The carbon atoms most directly involved in the early-time spectral shifts, C4 and C5, undergo significant angle displacement and bond rehybridization according to the TSH data (see Figure 3g–i and Figure 4g–i). This is also reflected in the *atom-resolved* TR-NEXAFS spectra, shown in Figure 9, according to which the early-time spectral shifts are mainly observed for C4 and C5.

**Figure 9:**
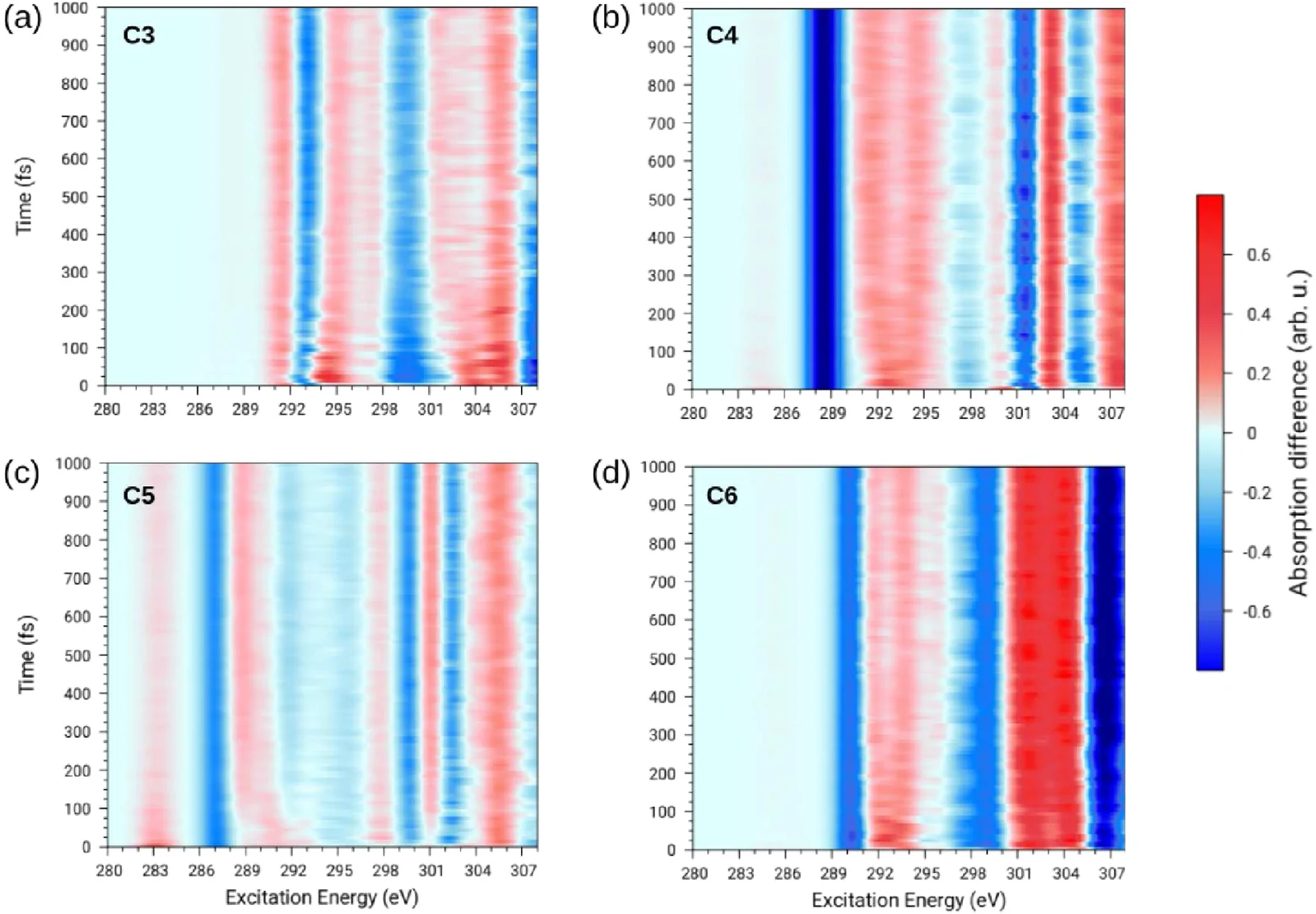
Atom-resolved TR-NEXAFS of the uracil molecule. (a), (b), (c) and (d) represent the C3, C4, C5 and C6 atom-resolved TR-NEXAFS of uracil, respectively. See Figure 1b for atom numbering.

In general, compared to O 1s and N 1s atom-resolved TR-NEXAFS, the atom-resolved C 1s TR-NEXAFS spectra are more diffuse and have more positive–negative absorption pairs. Due to its substantial spectral overlap, it is relatively difficult to assign unique site-specific signatures. Still, a few characteristic features can be observed from the C 1s atom-resolved spectrum. At lower photon energies (∼283 eV), the most prominent transient signal originates from the C5 site, which shows a strong positive feature that diminishes as the delay time increases. At the same energy, the C4 site also exhibits a weak positive contribution at ∼284 eV. These low-energy features are closely correlated with the structural dynamics of the C4–C5 double bond, as revealed by the bond length and bond angle changes observed in the SH simulations (see Figure 3g–i).

Strong ground-state bleach signals are observed for both C4 and C5. For C4, the bleach is centered at 289 eV, whereas for C5 it appears at slightly lower energies (∼287–288 eV). The C6 site also displays a pronounced bleach at 290–291 eV, along with a higher-energy bleach at 307 eV. In addition, C6 exhibits a broad and intense positive absorption band between 301 and 304 eV. The temporal evolution of the C5 spectrum reveals a notable spectral shift within the first 100–120 fs. Initially, a positive feature is observed at 293 eV, which gradually redshifts to ∼290 eV. Concurrently, the C5 feature at ∼301 eV increases in intensity. This coupled redshift and growth behavior is consistent with internal conversion dynamics occurring during this early-time window. In the same region (around 302 eV) the C3 spectrum also has some contributions: the negative features redshifts to 301 eV and the positive feature from 304 eV redshifts toward 301 eV.

## 4 Summary and Conclusions

In this paper, we presented an investigation of the early S_2_ → S_1_ relaxation dynamics of uracil using a combination of surface hopping and TR-NEXAFS simulations at the O 1s, N 1s, and C 1s edges. The simulations do not capture the subsequent recovery to the ground state within the simulated time window. For the S_2_ → S_1_ dynamics, we characterized the interplay between electronic relaxation and nuclear motion following UV photoexcitation, and established site-specific signatures of these processes through core-level spectroscopy.

Surface hopping dynamics initiated in the bright S_2_ (ππ*) state revealed a rapid and efficient internal conversion to the dark S_1_ (nπ*) state within the first ∼140 fs. No trajectories returned to the ground state (S_0_), which may be partially attributed to the nature of the underlying TD-DFT methodology. Nevertheless, the simulations captured the key aspects of excited-state dynamics, particularly the population trapping in S_1_ and the accompanying nuclear distortions. Detailed structural analyses revealed localized changes near the oxygen, nitrogen and ethylenic carbon centers, including shortening and lengthening of the carbonyl bond and asynchronous motion at the N1 and N2 sites as well as ring puckering associated with the C4–C5 bond.

Building on these dynamic structural results, we coupled our TR-NEXAFS simulations with the TSH calculations to capture how the local chemical environments evolve in real time across multiple atomic sites. At the oxygen edge, a pronounced asymmetry was observed between O7 and O8: O7 exhibited both a strong stationary peak at 529 eV and a dynamic, blue-shifting band (535 to 537 eV) correlated with C6–O7 bond elongation. In contrast, O8 showed minimal evolution, reflecting its limited role in the excited-state reorganization.

Nitrogen-resolved TR-NEXAFS revealed more diffuse features, indicative of overlapping state contributions. N2 showed gradual intensity decay at 399 eV, tracking S_2_ population loss, while N1 exhibited a pronounced change: the 414–417 eV absorption band diminished and a new feature emerged at ∼410 eV, indicating internal conversion (S_2_ → S_1_) at around 140 fs. These findings highlight the distinct and asynchronous involvement of the two nitrogen atoms during relaxation.

Finally, the carbon K-edge TR-NEXAFS spectrum shows rapid *π*-conjugation loss from torsional deformation of the pyrimidine ring resulted from C4 and C5 carbons within 25 fs at 282 eV. The 293 eV feature gradually weakens and simultaneously a new absorption feature emerges near 301 eV indicating internal conversion (∼140 fs) from the delocalized S_2_ (ππ*) to the more localized S_1_ (nπ*) state.

In summary, our TSH dynamics leave clear traces in the TR-NEXAFS spectra. In concert, TR-NEXAFS and nonadiabatic molecular dynamics give us a clear picture of coupled electron-nuclear dynamics after photoexcitation, enabling direct comparison with gas-phase TR-NEXAFS experiments in the future. In this work, for uracil, the combined approach reveals not only the key relaxation pathways but also the atomic sites most affected by photoinduced reorganization, offering an atom- and time-resolved understanding of ultrafast processes relevant to nucleobase photophysics. Inclusion of triplet states in TSH simulations in the context of TR-NEXAFS calculations should be addressed in the future research.

## Supplementary Material



## Data Availability

Data for this article are available at Zenodo at 10.5281/zenodo.19735475.

## Notes

Further results for C 1s TR-NEXAFS (PDF)
