# Supplementary material for: A Theoretical Investigation of Nonadiabatic Dynamics and Time-Resolved NEXAFS Spectra of Uracil
Source: ACS Omega. 2026 Sep 8;11(37):56251–64. doi: 10.1021/acsomega.6c06959 (PMC13613955; doi:10.1021/acsomega.6c06959)
Supplement: Supplementary file 1 [file ao6c06959_si_001.pdf]

Supporting Information for

A Theoretical Investigation of Nonadiabatic Dynamics and Time-Resolved NEXAFS Spectra of Uracil

Bonasree Roy, Evgenii Titov, Peter Saalfrank*

University of Potsdam, Institute of Chemistry, Theoretical Chemistry,

Karl-Liebknecht-Straße 24–25, 14476 Potsdam, Germany

*peter.saalfrank@uni-potsdam.de

August 16, 2026

Contents

S1 C 1s TR-NEXAFS **S2**

S1 C 1s TR-NEXAFS

The convergence of C 1s TR-NEXAFS spectrum was further assessed by comparing randomly selected subsets of 20 and 40 trajectories with the full ensemble of 56 trajectories. The three ensembles produce very similar spectral features, indicating that the main C 1s TR-NEXAFS (56 trajectories) signatures are well converged with respect to the number of trajectories.

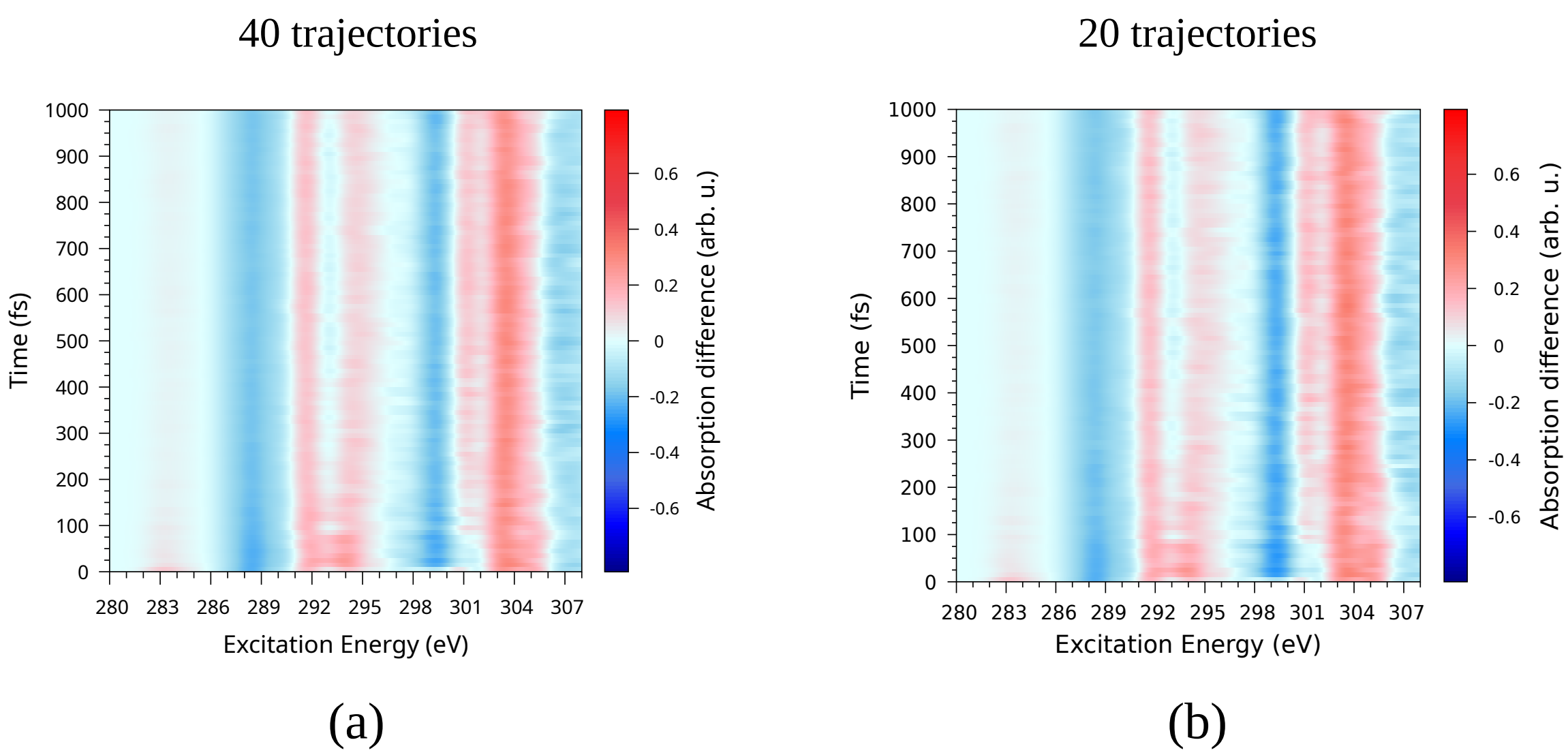

Figure S1: C 1s TR-NEXAFS of the uracil molecule with two subsets of 56 trajectory ensemble: (a) 40 trajectories, (b) 20 trajectories.
